# The impact of El Niño-Southern Oscillation on the incidence of infectious diarrhea in China: insights from a 15-year national surveillance analysis

**DOI:** 10.3389/fpubh.2026.1791469

**Published:** 2026-04-29

**Authors:** Keer Ou, Xing Li, Weilin Zeng, Yue Shi, Zuhua Rong, Yingtao Zhang, Shu Xiao, Zhongyi Fan, Mengjie Geng, Hongwei Tu, Jianpeng Xiao

**Affiliations:** 1 School of Public Health, Guangdong Pharmaceutical University, Guangzhou, China; 2 Guangdong Provincial Institute of Public Health, Guangdong Provincial Center for Disease Control and Prevention, Guangzhou, China; 3 National Key Laboratory of Intelligent Tracking and Forecasting for Infectious Diseases, Division of Infectious Disease, Chinese Center for Disease Control and Prevention (Chinese Academy of Preventive Medicine), Beijing, China; 4 Guangdong Provincial Center for Disease Control and Prevention, Guangzhou, China; 5 School of Medicine, Jinan University, Guangzhou, Guangdong, China; 6 School of Public Health, Southern Medical University, Guangzhou, Guangdong, China

**Keywords:** climate factor, El Niño Southern Oscillation, generalized additive model, infectious diarrhea, wavelet analysis

## Abstract

**Background:**

Infectious diarrhea remains a significant public health challenge, with climatic factors potentially playing a crucial role in its epidemiological spread. However, the precise mechanisms through which the El Niño-Southern Oscillation (ENSO) influences diarrheal morbidity are still not fully understood.

**Methods:**

We collected monthly other infectious diarrhea (OID) incidence and climatic data across the 31 provincial administrative divisions of mainland China (2005–2019). Wavelet analysis was employed to examine the periodicity of OID and the phase relationships between ENSO, climate factors and OID. Generalized additive models (GAM) and Peter and Clark Momentary Conditional Independence (PCMCI) algorithm were used to quantify exposure-response relationship and establish causal pathways in China.

**Results:**

From 2005 to 2019, a total of 13,620,167 OID cases were reported in 31 provincial regions of China, with the highest incidence of OID concentrated in the southern and eastern regions of China. Wavelet analysis identified a significant periodicity between ENSO cycles and diarrhea incidence patterns, demonstrating that La Niña events (characterized by low ENSO index) were associated with subsequent increases in incidence with a 6-month lag. The exposure-response relationship showed an inverted J-shaped curve in North and East China, while a nearly linear relationship was observed in Northeast, Central, Southwest and Northwest China. PCMCI analysis elucidated that precipitation is an indirect link between ENSO and OID.

**Conclusion:**

Our study suggests that a low ENSO index (La Niña) may drive the incidence of OID in China. The findings provide a scientific basis for predicting and warning of OID based on ENSO.

## 1 Introduction

Infectious diarrhea continues to pose a substantial global public health threat, particularly in developing nations (1). According to the World Health Organization (WHO), diarrheal diseases are the third leading cause of death among children under 5 years old, accounting for approximately 443,832 child fatalities annually, with nearly 1.7 billion cases reported each year (2). In China, other infectious diarrhea (OID), including Viral (e.g., norovirus and rotavirus gastroenteritis) and bacterial gastroenteritis (e.g., *Escherichia coli* and Shigella infection), is most prevalent among children aged <5 years, with an annual incidence rate of 60 cases per 100,000 children (3). The epidemiology of OID exhibits distinct seasonal patterns and regional variations, typically peaking during the summer and autumn months (4, 5). Evidence suggests that recent climatic variability and extreme weather events may influence OID transmission (6), and bacterial and viral pathogens constitute the predominant etiological agents (7).

Climatic factors significantly affect diarrheal disease transmission (8). The El Niño-Southern Oscillation (ENSO) is a natural climate phenomenon characterized by periodic fluctuations in sea surface temperatures(SST) and atmospheric pressure across the tropical Pacific Ocean (9), which indirectly affects diseases by altering regional climatic patterns (10). A study showed that a 12-month-lagged ENSO index was associated with an increased risk of dengue fever in Guangdong Province (11). Research in Korea showed that the La Niña events may lead to an increased incidence of diarrheal disease (12). While previous studies have explored the association between ENSO and infectious diarrhea (13), these investigations have not specifically focused on OID. Meanwhile, a growing body of research has begun to examine the impact of other climatic factors, such as ambient temperature and sunshine duration, on the incidence of OID (14, 15). Although several climatic factors have been examined in OID research, the impact of the ENSO on OID remains unclear and warrants further investigation.

This study aims to elucidate the mechanisms underlying ENSO-driven transmission of OID by examining the epidemiological characteristics of OID in China from 2005 to 2019 and their associations with ENSO events. By using wavelet analysis, generalized additive models (GAM), and Peter and Clark Momentary Conditional Independence (PCMCI) causal inference methods, we examine the exposure-response relationship between ENSO and the incidence of OID and elucidate the factors such as temperature and precipitation is an indirect link between ENSO and OID.

## 2 Methods

### 2.1 Data collection

#### 2.1.1 Study area and incidence of OID

The data in this study covered the 31 provincial administrative divisions of mainland China, including 22 provinces, 5 municipalities and 4 autonomous administrative regions (hereafter referred to as 31 provinces). Based on China’s geographical environment, climate, economy and other factors, the 31 provinces can be divided into seven major regions: North China, East China, South China, Southwest China, Northwest China, Northeast China, and Central China (16). The regions are illustrated in Supplementary Figure S1. Hong Kong, Macao, and Taiwan were not included due to data unavailability.

According to the Diagnostic Criteria for Infectious Diarrhea (WS 271–2007) issued by the Ministry of Health of the People’s Republic of China, Other Infectious Diarrhea (OID) is defined as infectious diarrhea caused by pathogens other than cholera, dysentery, typhoid, or paratyphoid, which is categorized as a Class C notifiable infectious disease. We obtained the monthly OID cases and incidence rates per 100,000 population from January 2005 to December 2019 from the Public Health Science Data Center, which provided us with the information, including administrative divisions and incidence rates. The provincial data were standardized and subsequently aggregated into the OID data of seven regions.

#### 2.1.2 Meteorological data and ENSO index

Monthly meteorological data were obtained from the China Meteorological Science Data Center from 2005 to 2019, covering 179 monitoring stations across 31 provinces in China. The variables included mean temperature (°C), mean atmospheric pressure (hPa), mean precipitation (mm), mean relative humidity (%), and mean wind speed (m/s). Provincial-level data were derived by averaging measurements from all monitoring stations within each province. Missing values were filled using the monthly mean of the preceding and following months.

The ENSO index used the Oceanic Niño Index (ONI), which is the sea surface temperature anomalies in the Niño region 3.4 (5°S–5°N, 170°E–120°W), sourced from the Climate Prediction Center of the U. S. National Weather Service. After compiling monthly meteorological data and ENSO index data for all provinces (2005–2019), these were matched with the incidence of OID using provinces and incidence date, and aggregated into the OID data of seven regions for subsequent analysis.

### 2.2 Statistical analyses

#### 2.2.1 Wavelet analysis

Wavelet analysis was employed to examine the periodicity of OID, the phase relationship between ENSO, local weather, and OID. To reduce skewing between variables in wavelet analysis, the incidence of OID in seven regions of China was square-root transformed, and both the ENSO index and incidence data were standardized (17). Morlet wavelet transform was applied to generate wavelet power spectra for ENSO and incidence of OID, enabling identification of their periodic characteristics. Continuous Wavelet Transform (CWT) decomposes a time series by translating and scaling a series of wavelet functions across different scales, effectively identifying the periodicity of dominant oscillations within the time domain. The specific formula is as Equation 1:


$$\text{Wave}(T,s)=\sum_{t}x_{t}\frac{1}{\sqrt{s}}\psi^{*}(\frac{t-T}{s})$$ (1)

t is the time parameter, and s is the scale parameter, representing the frequency of the waveform for each series under investigation. $x_{t}$ denotes the studied time series, which in this study includes the monthly incidence of OID and ENSO index. $\psi^{*}$ is the complex conjugate of the Fourier-transformed mother wavelet function ψ(x). This study applies the widely used continuous wavelet transform to analyze the time series of OID incidence and the ENSO index. The generated wavelet power spectrum and its statistical significance testing reveal the temporal evolution and periodic characteristics of the series.

Cross-wavelet transform was employed to examine co-variability and reveal their phase association between the two time series (18). The cross-wavelet transform (XWT) examines the interrelationship between two time series in the time-frequency domain across multiple time scales, and can effectively analyze the correlation, time lag, and phase structure between them. The formula for wavelet coherence is as Equation 2:


$$R_{x,y}(f,s)=\frac{{|W_{\mathrm{xy}}(f,s)|}^{2}}{|W_{x}(f,s)\|W_{y}(f,s)|}$$ (2)

$W_{\mathrm{xy}}(f,s)\;$ is the cross-wavelet transform of time series x(t) and y(t) , representing their point-wise product, which measures the interaction between the two signals at a specific frequency f and scale s . $\;W_{x}(f,s)\;$ and $\;W_{y}(f,s)\;$ describe the characteristics of each individual signal. The squared modulus $\;{|W_{\mathrm{xy}}(f,s)|}^{2}$ quantifies the co-variation energy of the two signals at the given frequency and time scale. The wavelet coherence $\;R_{x,y}(f,s)$ is a dimensionless index ranging between 0 and 1. It indicates the degree of linear correlation between the two time series at a specific frequency and time scale.

Subsequently, the cross-wavelet phase angle is used to describe the local phase relationship between time series x and y in the time-frequency domain. The specific formula is as Equation 3:


ϕx,y(f,s)=tan−1Im(Wxy(f,s))Re(Wxy(f,s)) (3)

$\phi_{x,y}(f,s)\;$ represents the phase difference between the two time series at a specific frequency f and scale s, The arctangent function, $\;\tan^{-1}$ , calculates this phase difference from the real and imaginary parts of the cross-wavelet transform. If the phase difference is close to 0 or an integer multiple of 2π . The phase difference is then used to calculate the phase angle, which, in combination with the periodic value of the time series, allows for the computation of the time lag between the two sequences. In this study, the time lag between ENSO, local weather, and the incidence of OID cycles was quantified by computing phase angles and periods.

#### 2.2.2 Cross-correlation analysis

Cross-correlation analyses with 1-, 2-, and 3-month lags were conducted between the incidence of OID and the ENSO index, mean precipitation, temperature, relative humidity, wind speed, atmospheric pressure, maximum temperature, and minimum temperature to explore the correlations between meteorological factors and the incidence of OID.

#### 2.2.3 GAM analysis

GAM is particularly suited for capturing nonlinear relationships between meteorological factors and health outcomes, and has been widely applied in environmental epidemiology and public health research (19–21). To further examine the exposure-response relationship between ENSO and the incidence of OID in seven regions of China, we performed GAM analysis using monthly data. We employed cubic spline functions for the predictor variables and used a quasi-Poisson model to account for over-dispersion in the case count data. The model was specified as Equation 4:


$$\begin{array}{l} \log(u_{t})=\beta_{0}+s(\text{Variabl}e_{t-e},\mathrm{df})+s(\text{month},\mathrm{df})\\ +\mathrm{as}.\text{factor}(\text{year}) \end{array}$$ (4)

The model specifies $u_{t}$ as the diarrhea case count in month t , with climate variables modeled using cubic splines incorporating lags up to e months. The lag was selected according to the phase difference results from the wavelet analysis of the seven regions in China (22). The lag of the ENSO index was set to the integer-rounded value of the mean phase difference (months) for each region, which derived from the wavelet cross-analysis (Tables 1, 2). The lagged months of other controlled variables such as temperature, precipitation, humidity, and wind speed are shown in Table 2. The highly correlated mean temperature and mean precipitation were assigned to separate models to avoid multicollinearity, and relative humidity and wind speed were controlled as control variables for the GAM model. The final model and the degrees of freedom (df) for the month variable were selected by minimizing the Generalized Cross Validation (GCV) score. The df for ENSO and other meteorological factors was set to 3. We adjusted the monthly df within the range of 5–7, and the df of meteorological factors within the range of 3–5 for sensitivity analysis.

**Table 1:** Phase differences between ENSO index and other infectious diarrhea (OID) incidence in overall China and its seven regions during 2005–2019.

Region	Angle difference	Mean difference (months)
Overall China	31.41°	5.2
North China	41.20°	6.9
Northeast China	26.90°	4.5
East China	29.04°	4.8
Central China	50.64°	8.4
South China	25.15°	4.2
Southwest China	28.36°	4.7
Northwest China	30.29°	5.0

**Table 2:** Lag month between climatic factors and other infectious diarrhea (OID) incidence in overall China and its seven regions during 2005–2019.

Region	Lag month				
	ENSO	Temperature	Precipitation	Humidity	Wind speed
Overall China	5	2	2	1	3
North China	7	1	1	1	4
Northeast China	6	1	1	1	4
East China	5	2	2	1	5
Central China	8	2	2	1	4
South China	4	3	3	1	3
Southwest China	5	2	2	2	1
Northwest China	5	1	1	1	3

#### 2.2.4 PCMCI analysis

We employed the PCMCI method to investigate causal relationships between ENSO and the incidence of OID rate in seven regions of China. PCMCI is a causal discovery method for time-series data that identifies directed relationships while controlling for auto-correlation and high dimensionality. The method selects relevant conditions via iterative independence tests (PC step), then applies momentary conditional independence tests (MCI step) to establish causality (23).

The PCMCI framework operates within the graphical causal model framework. Consider a set of N observed time series variables $X_{t}=(X_{t}^{1},\ldots ,X_{t}^{N})$ . The underlying dynamical system is assumed to follow Equation 5:


$$X_{t}^{j}=f_{j}(P(X_{t}^{j}),\eta_{t}^{j})$$ (5)

$f_{j}$ is a potentially nonlinear function, $\eta_{t}^{j}\;$ is noise, and $P(X_{t}^{j})\subset X_{t}^{-}=(X_{t-1},X_{t-2},\ldots )$ denotes the causal parents of variables $X_{t}^{j}$ . The PCMCI algorithm proceeds in two stages:

In Condition Selection (PC stage), the algorithm starts with *p* = 0, within an outer loop, the size of the conditioning set is iteratively increased (p → p + 1). In the inner loop, for all variables $\;X_{t}^{j}\;$ from $\hat{\;P}(X_{t}^{j})\;$ , the procedure (Equation 6) tests whether the conditional independence null hypothesis:


$$\mathrm{PC}:X_{t-\tau }^{i}∐X_{t}^{j}/S\;\text{for}\;\mathrm{any}\;\;S\;\text{with}/S/=p$$ (6)

Can be rejected at the significance level $\alpha_{\mathrm{PC}}$ . This step drastically reduces the dimensionality of the conditioning set, thereby increasing the statistical power to detect true links.

In Causal Link Testing (MCI Stage), the Momentary Conditional Independence (MCI) test is applied to each potential causal link $X_{t-\tau }^{i}\to X_{t}^{j}$ . The test assesses (Equation 7) under the following null hypothesis:


$$X_{t-\tau }^{i}∐X_{t}^{j}/\hat{P}(X_{t}^{j})\backslash \{X_{t-\tau }^{i}\},\hat{P}(X_{t-\tau }^{i})$$ (7)

The parents of the lagged source variable $X_{t-\tau }$ statistically significant if the MCI test rejects the null hypothesis at a significance level of alpha_level = 0.05.

Mean temperature and precipitation were selected for causal analysis as the meteorological factors most strongly correlated with the incidence of OID rate (Supplementary Figure S3). For the conditional independence tests in the PC step, pc_alpha was set to 0.05 for all regional models. The maximum lag time is determined by the wavelet analysis lag time (Table 2), and the alpha_level was set as 0.05 at the MCI step. The environmental and climatic factors used in this study are listed in Table 3.

**Table 3:** Climatic parameters used for analysis of OID incidence.

Parameter	Analysis		
	Basic information	GAM	PCMCI
ENSO	√	√	√
Mean Temperature	√	√(covariate)	√(proximal indicator)
Precipitation	√	√(covariate)	√(proximal indicator)
Relative humidity	√	√(covariate)	
Wind speed	√	√(covariate)	
Atmospheric pressure	√		
Maximum temperature	√		
Minimum temperature	√		

In the present study, Wavelet analysis and GAM analysis were performed using the “WaveletComp” and “mgcv” packages in R software version 4.3.0. We use the “Tigramite” package in Python, which allows performing PCMCI from time series.

## 3 Results

### 3.1 Basic information

**Figure 1:**
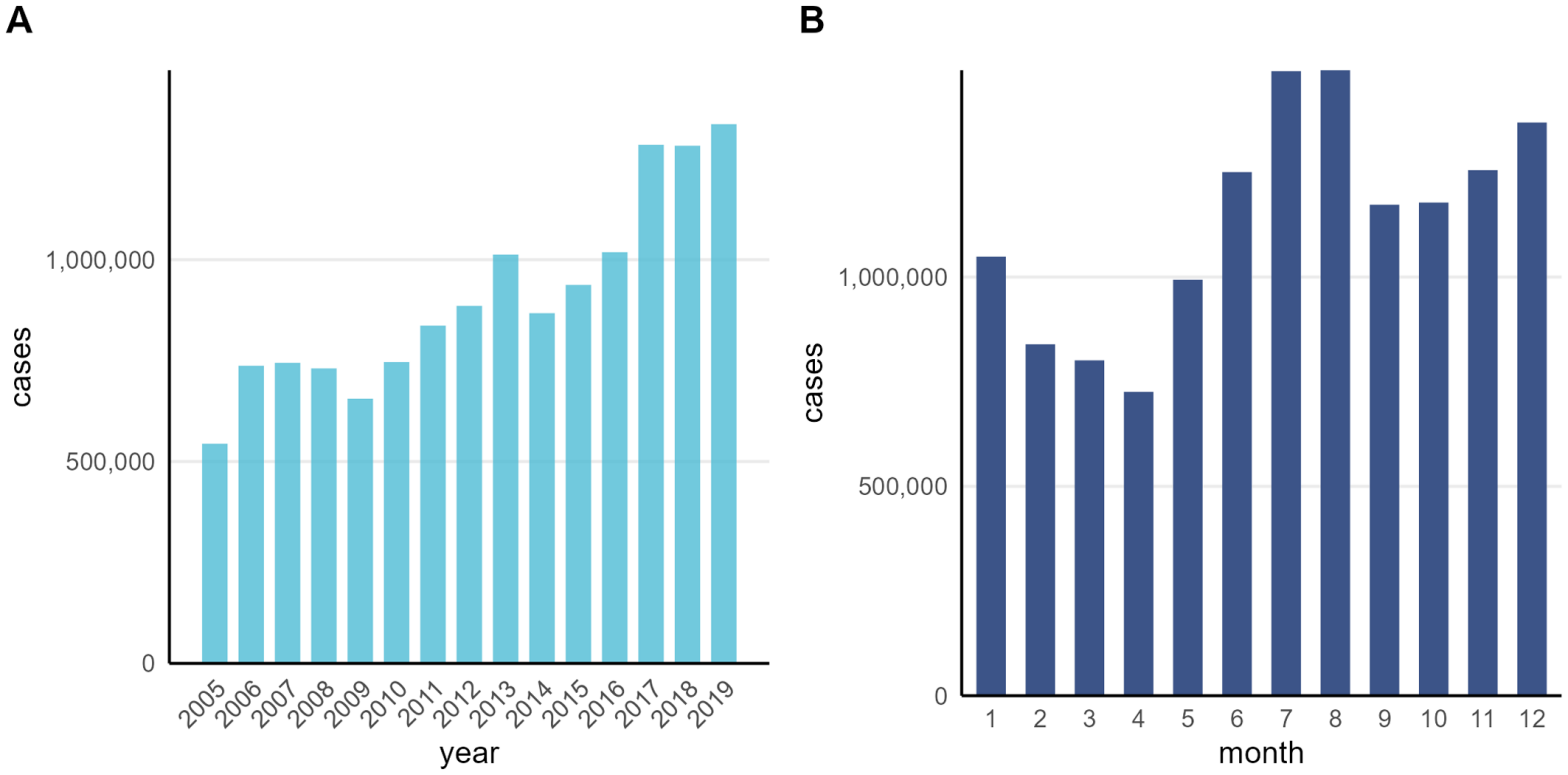
Annual and monthly distribution of other infectious diarrhea (OID) cases in China, 2005–2019 **(A)** annual distribution of OID; **(B)** monthly distribution of OID.

**Figure 2:**
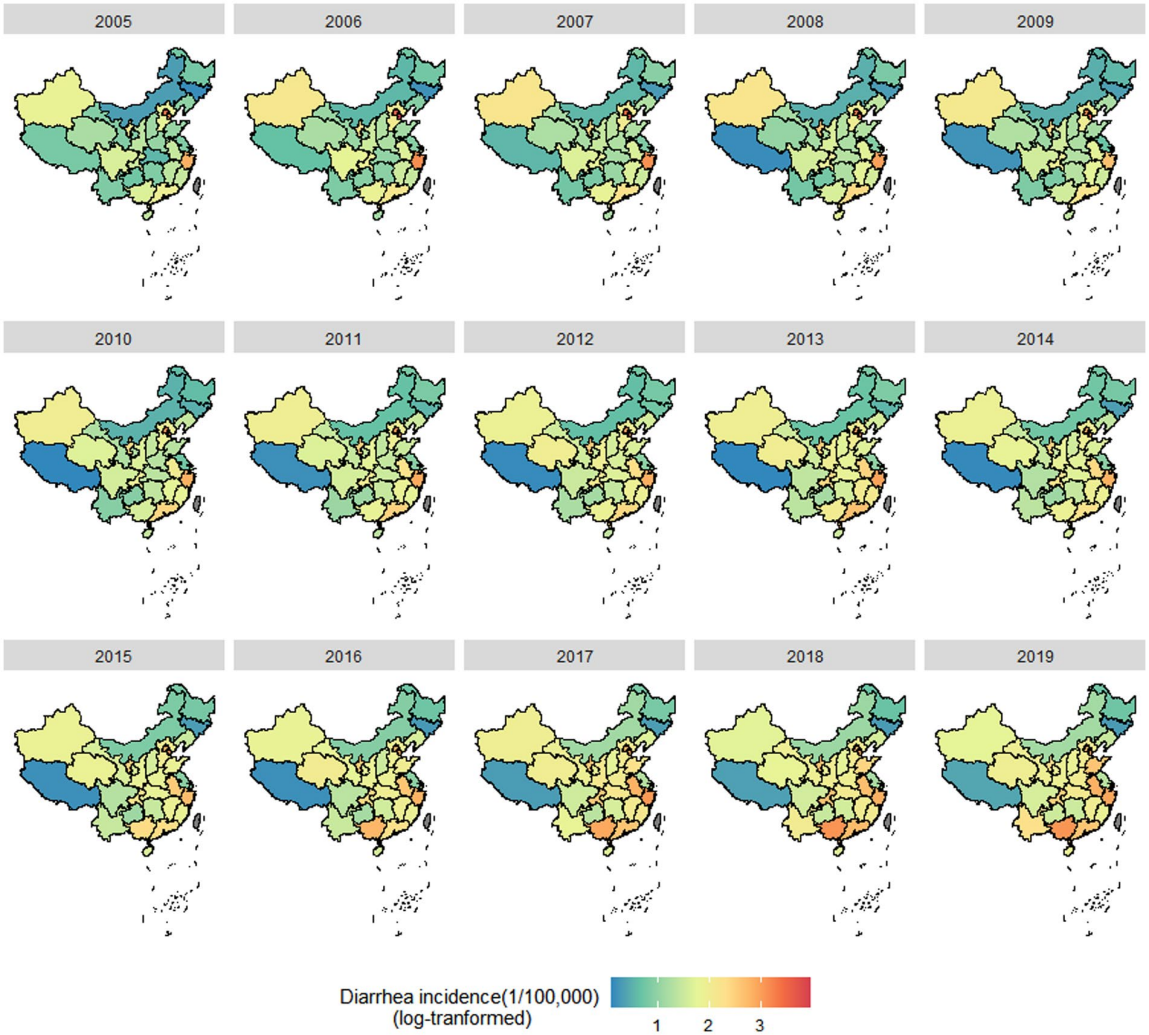
Spatial distribution of the incidence of other infectious diarrhea (OID) in China, 2005–2019.

A total of 13,620,167 cases of OID were reported in mainland China during 2005–2019. The peak national incidence occurred in 2019, with a total of 1,335,627 cases reported. The basic epidemiological and meteorological characteristics of monthly cases and climate variables for all 31 provinces are presented in Table 4. The average annual number of OID cases showed an increasing trend from 2005 to 2019 (Figure 1A). Figure 1B illustrates the cumulative monthly case numbers of OID from 2005 to 2019. OID exhibited a bimodal seasonal pattern with a primary peak occurring between June and August and a secondary peak from November to January of the following year. The overall incidence of OID varied with an increasing trend. The highest incidence of OID was concentrated in South China and East China regions spatially (Figure 2).

**Table 4:** Basic characteristics of the monthly incidence of OID and climatic factors in 31 provinces in China, 2005–2019.

Variables	Mean ± SD	Min	P25	Median	P75	Max
Number of cases	75667.59 ± 29786.81	16,411	54,880	76,078	96,428	183,493
ENSO (°C)	−0.10 ± 0.83	−1.6	−0.6	−0.15	0.5	2.6
Mean precipitation (mm)	76.70 ± 85.38	0.00	13.34	48.17	112.45	1066.93
Mean atmospheric pressure (hPa)	943.00 ± 97.10	646.60	909.95	992.50	1006.45	1032.40
Mean temperature (°C)	13.76 ± 10.83	−22.62	6.50	15.44	22.50	32.00
Mean relative humidity (%)	64.87 ± 14.41	13.60	55.49	68.00	76.37	91.42
Mean maximum temperature (°C)	19.28 ± 10.42	−16.88	12.00	21.20	27.67	36.40
Mean minimum temperature (°C)	9.43 ± 11.32	−27.49	2.20	10.97	18.25	28.20
Mean wind speed (m/s)	2.15 ± 0.55	0.8	1.75	2.08	2.48	4.30

### 3.2 Wavelet analysis

#### 3.2.1 Periodicity of OID and ENSO

Wavelet analysis reveals distinct periodicity in the incidence of OID in China. In mainland China, the annual cycles of monthly OID incidence (pre-2015) transitioned to semi-annual cycles (post-2015). The ENSO index exhibits periodicity on a 1–8 year scale, with the dominant periodicity occurring at a 3–4 year scale (Figure 2).

#### 3.2.2 Cross-correlation and phase differences

**Figure 3:**
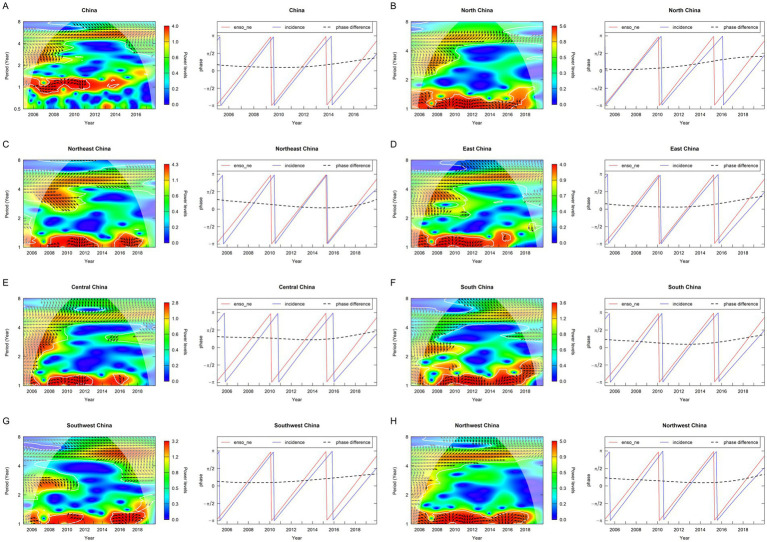
Wavelet coherence and phase-difference patterns of the ENSO index and the incidence of OID in overall China and the seven regions during 2005–2019 (5-year cycle) **(A)** Overall China; **(B)** North China; **(C)** Northeast China; **(D)** East China; **(E)** Central China; **(F)** South China; **(G)** Southwest China; **(H)** Northwest China. ENSO_ne, the negative phase of the ENSO index.

The cross-wavelet analysis between ENSO and the incidence of OID revealed significance across the 1–8-year scale range during 2005–2019. Phase arrows predominantly pointing to the upper right indicated that La Niña events (characterized by a low ENSO index) lag approximately 6 months and were associated with an increase in the incidence of OID. Analysis of the seven major regions was consistent with the nationwide results, demonstrating statistically significant periodicities at both 1-year and 3–6 year scales. Phase difference analysis demonstrated that low ENSO index preceded the increase of OID incidence with a half-year lag (30°phase difference; Figure 3). Mean temperature and precipitation were associated with the incidence of OID at 1–3 months lags (Supplementary Table S1).

### 3.3 Exposure-response relationship analysis

**Figure 4:**
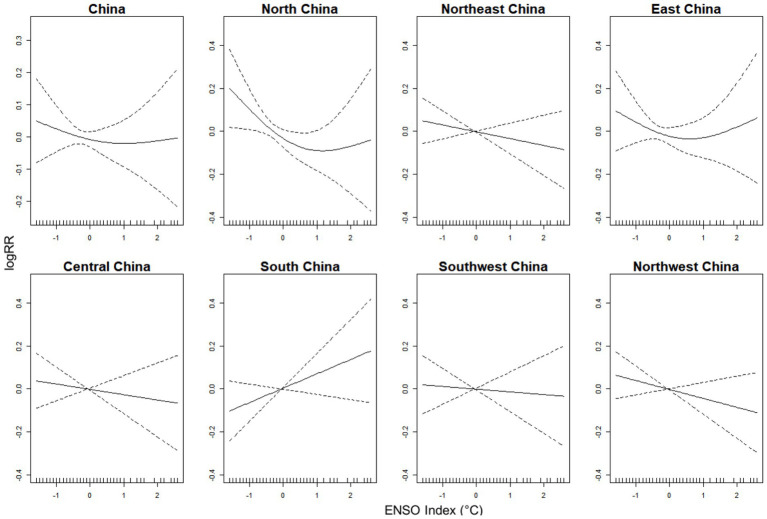
The exposure-response curves between the ENSO index and the incidence of other infectious diarrhea (OID) in overall China and its seven regions, 2005–2019.

The results of cross-correlation analyses indicated that both mean temperature and precipitation were moderately correlated with the ENSO index and were strongly positively correlated with each other (Supplementary Figure S3). Based on the GCV criterion, mean precipitation, relative humidity, and wind speed were selected as control variables for the GAM model. The results showed that the exposure-response curves for North and East China exhibited an inverted J-shaped relationship. In contrast, Northeast, Central, Southwest, and Northwest China displayed a nearly linear relationship, where lower ENSO indices were associated with a higher incidence of OID. However, the curve for South China suggested a positive linear correlation between the El Niño and the incidence of OID (Figure 4). Supplementary Table S3 illustrates the effect of ENSO index on the incidence of OID. In Northeast China, the estimated coefficients showed negative values. It is estimated that for every 1-unit decrease in the ENSO index, the log incidence rate of OID, on average, increased by 0.032 units. Sensitivity analysis suggested that the results of the GAM model were robust (Supplementary Figures S4, S5).

### 3.4 Causal relationships derived from PCMCI analysis

**Figure 5:**
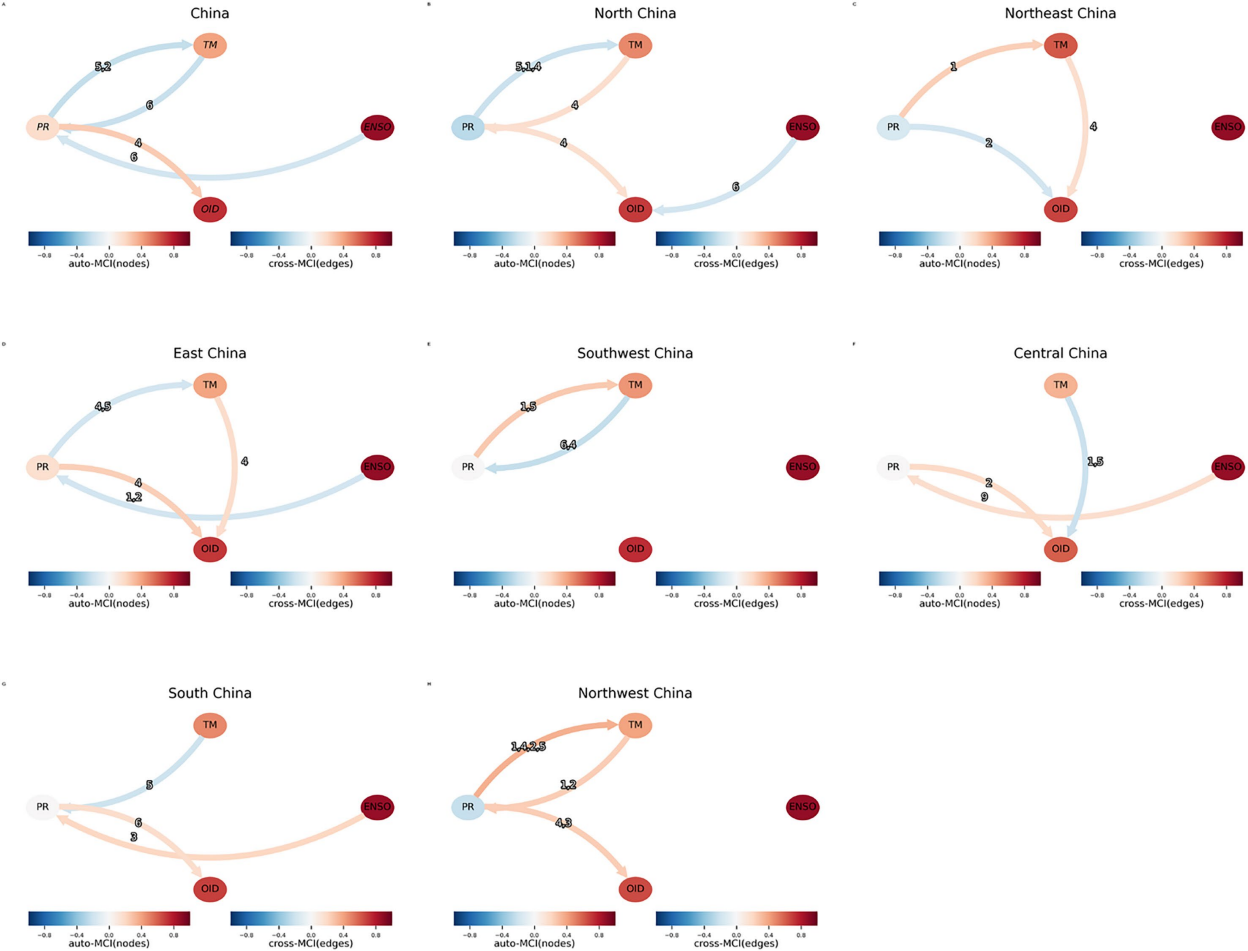
PCMCI analysis of temperature (TM), precipitation (PR), ENSO index (ENSO), and incidence of other infectious diarrhea (OID) in seven regions in China, 2005–2019. **(A)** Overall China; **(B)** North China; **(C)** Northeast China; **(D)** East China; **(E)** Southwest China; **(F)** Central China; **(G)** South China; **(H)** Northwest China. MCI, Momentary conditional independence; auto-MCI, autocorrelation strength by node color; cross-MCI, causal strength by edge color.

Results of the PCMCI indicated that the ENSO index exerted both direct and indirect pathways influences on the incidence of OID in China (Figure 5). The ENSO events primarily influenced the incidence of OID by modulating the mean precipitation, which revealed that mean precipitation is an indirect link between ENSO and OID.

In China, the ENSO index at a 6-month lag exhibited a negative causal relationship with the mean precipitation, while the mean precipitation at a 4-month lag exhibited a positive causal relationship with the incidence of OID. This result indicated that the low ENSO index at a 6-month lag indirectly influenced the incidence of OID through the mean precipitation as an indirect link variable in China. A direct negative causal link was observed between the 6-month-lagged ENSO index and the incidence of OID in North China. In East China, ENSO at 1- and 2-month lags negatively influenced the mean precipitation. Positive causal relationships between the ENSO index and mean precipitation were observed in Central and South China. Notably, except for Southwest China, mean precipitation in Northeast China exerted a negative causal effect on the incidence of OID, while mean precipitation at varying lag times exhibited positive causal associations with the incidence of OID in other regions.

## 4 Discussion

This study analyzed the epidemiological characteristics and explored the impact of climatic factors on OID in China. We observed that OID exhibits seasonal fluctuations, which are associated with climatic variables and ENSO. Furthermore, the lagged occurrence of La Niña events influenced the incidence of OID in seven regions of China, revealing notable regional variations. This study has provided evidence that La Niña events several months prior showed an impact on increasing OID incidence, potentially indirect linked through precipitation. These findings provide a scientific basis for using the ENSO index to assess risks and issue early warnings for OID epidemic.

### 4.1 Epidemiological characteristics of OID

Our study observed a gradual increase in the incidence of OID, potentially due to enhancements in disease surveillance systems (24), increased population mobility (25), climate change (26), or pathogen evolution (27, 28). The distinct seasonality of OID may be attributed to warm, humid summers favoring bacterial spread, and winter conditions favoring viral transmission (29). Spatially, the incidence of OID was highest in South and East China, which is consistent with a study (25). These areas experience substantial population mobility, which may further increase transmission risks (30). Wavelet periodicity analysis revealed the periodicity of the incidence of OID, consistent with numerous contemporary studies (31–33) and further supported by our descriptive findings.

### 4.2 The impact of ENSO on OID

We found that the La Niña events (low ENSO index) precede the incidence of OID in China. Cross-wavelet analysis revealed that La Niña events (low ENSO index) correlated with diarrhea incidence at a 6-month lag, consistent with findings from Botswana, Africa (0–5 month lags for diarrhea) (34). However, the lagged months exhibited divergence from a study conducted in Shandong, China (35). La Niña events are associated with increased diarrhea incidence, likely by altering climatic factors such as temperature and precipitation (36, 37). The results of the exposure-response relationship between the ENSO index and diarrhea agree with the results of the study in Nepal (38) and a study in the Yangtze River Basin, China (39). However, a study in Peru found that moderate-to-strong El Niño events, but not La Niña, were associated with increased diarrhea incidence among children under 5 years old (40). These discrepancies may be attributed to regional climate variations induced by El Niño and La Niña events, which indirectly influence diarrhea transmission pathways. Our findings suggested that El Niño events in South China may lead to an increase in the incidence of OID, indicating that the influence of ENSO on OID exhibits regional specificity, and there may be more underlying factors affecting the impact of ENSO on OID. The local climatic response to ENSO phases in South China may be distinct. El Niño events can alter regional precipitation (41), and the increased extreme rainfall or higher humidity during El Niño phases, which might be conducive to pathogen transmission in South China (29). The regional pathogen profile could be a risk factor (42). Our study did not differentiate between viral and bacterial diarrhea (7, 28), and if the OID in South China is dominated by pathogens which is more sensitive to El Niño-driven weather patterns, this could explain the divergent exposure-response relationship. Geographic and socio-demographic factors unique to this coastal, densely populated region with high population mobility (30) may influence the OID incidence, potentially amplifying transmission risks during specific ENSO phases. These results underscore the region-specific nature of ENSO and infectious diarrhea, necessitating risk assessments incorporating local pathogen profiles and sanitation infrastructure.

### 4.3 Lag effect and causal association between ENSO and OID

The influence of climate factors on diseases generally has a certain lag. Phase difference analysis in our study revealed distinct regional and temporal heterogeneity between ENSO and the incidence of OID in China, with varying lag times observed in different regions. Furthermore, our study identified a 1–3 month lag effect of precipitation and temperature on the incidence of OID, consistent with findings from previous studies (43, 44). Compared to the lagged effects of La Niña on OID, precipitation and temperature exhibit shorter lag times. Studies indicated that La Niña affected meteorological variables such as precipitation and temperature (40, 41, 45), and these climate factors also impacted the incidence of OID (46, 47). Thus, La Niña may indirectly influence OID rates through an indirect causal pathway linked by precipitation and temperature. PCMCI analysis revealed causal relationships between ENSO and OID incidence, showing that La Niña may indirectly influence OID rates through indirect pathways linked by precipitation and temperature. La Niña events are generally associated with increased precipitation in eastern and southern China. For instance, a study has observed an increase in summer rainfall during La Niña years, leading to substantially increased precipitation in specific regions (48). Another study revealed that intense precipitation can significantly elevate the transmission risk of OID (45). The increased rainfall can potentially increase the risk of OID. The possible reason is that excessive rainfall may cause flooding, which can contaminate drinking water sources, and the humid environment is conducive to the survival and spread of enteric viruses, such as norovirus and rotavirus. These findings suggest that ENSO may influence the incidence of OID through meteorological factors such as precipitation. However, as the analysis relied solely on PCMCI for exploratory causal inference, future research should incorporate multiple causal inference methods (e.g., Granger Causality Test, Convergent Cross Mapping) for comparative validation. Additionally, more in-depth mechanistic investigations are necessary to elucidate the pathway between ENSO and OID. Following this, the mediation effect within this pathway requires subsequent verification and quantification.

### 4.4 Strengths and limitations

To our knowledge, this is one of the few studies to investigate the association and the lagged effects between ENSO and the incidence of OID with regional levels in China, and to preliminarily explore potential causal links between temperature, precipitation, ENSO and OID. This study has several limitations. First, we did not distinguish between viral and bacterial diarrhea across seven regions, which may obscure pathogen-specific effects of ENSO. Second, the monthly incidence data limited the sample size for time-series analysis, potentially leading to unstable model estimates. Finally, the lack of control of socioeconomic and behavioral data, such as handwashing, water treatment, access to healthcare, could confound the observed associations between ENSO and the incidence of OID.

## 5 Conclusion

Our study revealed the low ENSO index (La Niña) being linked to a nationwide rise in diarrhea with a lag of 4–8 months, and the relationship varied in seven regions of China. This ENSO-driven risk pattern offers a practical tool for anticipating outbreaks of diarrhea and can inform evidence-based public-health policy.

## Data Availability

The datasets presented in this study can be found in online repositories. The names of the repository/repositories and accession number(s) can be found at: the data of the monthly OID cases and incidence rates can be obtained from the Public Health Science Data Center: https://www.phsciencedata.cn/Share/. Monthly meteorological data were obtained from the China Meteorological Data Service Centre: http://data.cma.cn/. The Oceanic Niño Index is derived from the National Oceanic and Atmospheric Administration (NOAA) Climate Prediction Center of NOAA/National Weather Service: https://origin.cpc.ncep.noaa.gov/.
