# Supplementary material for: The impact of El Niño-Southern Oscillation on the incidence of infectious diarrhea in China: insights from a 15-year national surveillance analysis
Source: Front Public Health. 2026 Apr 29;14:1791469. doi: 10.3389/fpubh.2026.1791469 (PMC13168187; doi:10.3389/fpubh.2026.1791469)
Supplement: Supplementary file 1 [file Data_sheet_1.docx]

Supplementary Material

Table S1 Phase differences between meteorological factors and the incidence of OID in seven regions of China, 2005–2019.

|  | **Low ENSO index** | | **Temperature** | | **Precipitation** | |
| --- | --- | --- | --- | --- | --- | --- |
|  | **Angle difference** | **Mean difference (days)** | **Angle difference** | **Mean difference (days)** | **Angle difference** | **Mean difference (days)** |
| China | 29.99° | 152 | 71.90° | 73 | 75.49° | 77 |
| North China | 27.41° | 139 | 17.96° | 18 | 7.68° | 8 |
| Northeast China | 21.57° | 109 | 10.11° | 10 | 7.23° | 7 |
| East China | 24.86° | 126 | 47.97° | 49 | 72.55° | 73 |
| Central China | 51.23° | 259 | 60.62° | 61 | 73.29° | 74 |
| South China | 33.76° | 171 | 79.54° | 81 | 76.37° | 77 |
| Southwest China | 30.70° | 156 | 56.42° | 57 | 56.63° | 57 |
| Northwest China | 27.67° | 140 | 42.02° | 42 | 29.92° | 30 |

**Table S2** **Generalized cross validation of GAM models for different monthly degrees of freedom in seven regions of China, 2005–2019. (with fixed df=3 for other variables)**

| **Models** |  | **GCV** | | | | | | | |
| --- | --- | --- | --- | --- | --- | --- | --- | --- | --- |
|  | **Month**  **df** | **China** | **North China** | **Northeast China** | **East China** | **Central China** | **South China** | **Southwest China** | **Northwest China** |
| log[$u_{t}$]=α+s(ENSO)  +s(temperature)  +s(relative humidity)  +s(wind speed)  +s(month) +as.factor(year) | 5 | 0.324 | 0.691 | 0.088 | 0.552 | 0.233 | 0.585 | 0.245 | 0.348 |
|  | 6 | 0.315 | 0.695 | 0.089 | 0.548 | 0.218 | 0.586 | 0.247 | 0.345 |
|  | 7 | 0.315 | 0.700 | 0.086 | 0.550 | 0.217 | 0.586 | 0.247 | 0.339 |
| log[$u_{t}$]=α+s(ENSO)  +s(precipitation)  +s(relative humidity)  +s(wind speed)  +s(month) +as.factor(year) | 5 | 0.317 | 0.684 | 0.089 | 0.546 | 0.235 | 0.549 | 0.243 | 0.350 |
|  | 6 | 0.307 | 0.689 | 0.090 | 0.545 | 0.224 | 0.545 | 0.245 | 0.350 |
|  | 7 | 0.304 | 0.693 | 0.088 | 0.546 | 0.221 | 0.546 | 0.245 | 0.339 |

Table S3 Estimate (β) of ENSO and the incidence of OID in seven regions of China, 2005–2019.

| **Region** | **Model shape** | **Edf** | **Estimate (β)** |
| --- | --- | --- | --- |
| China | Nearly inverted J-shaped | 1.306 | - |
| North China | Inverted J-shaped | 1.618 | - |
| Northeast China | Linear | 1.000 | -0.032 |
| East China | U-shaped | 1.541 | - |
| Central China | Linear | 1.000 | -0.026 |
| South China | Linear | 1.000 | 0.067 |
| Southwest China | Linear | 1.000 | -0.013 |
| Northwest China | Linear | 1.000 | -0.041 |


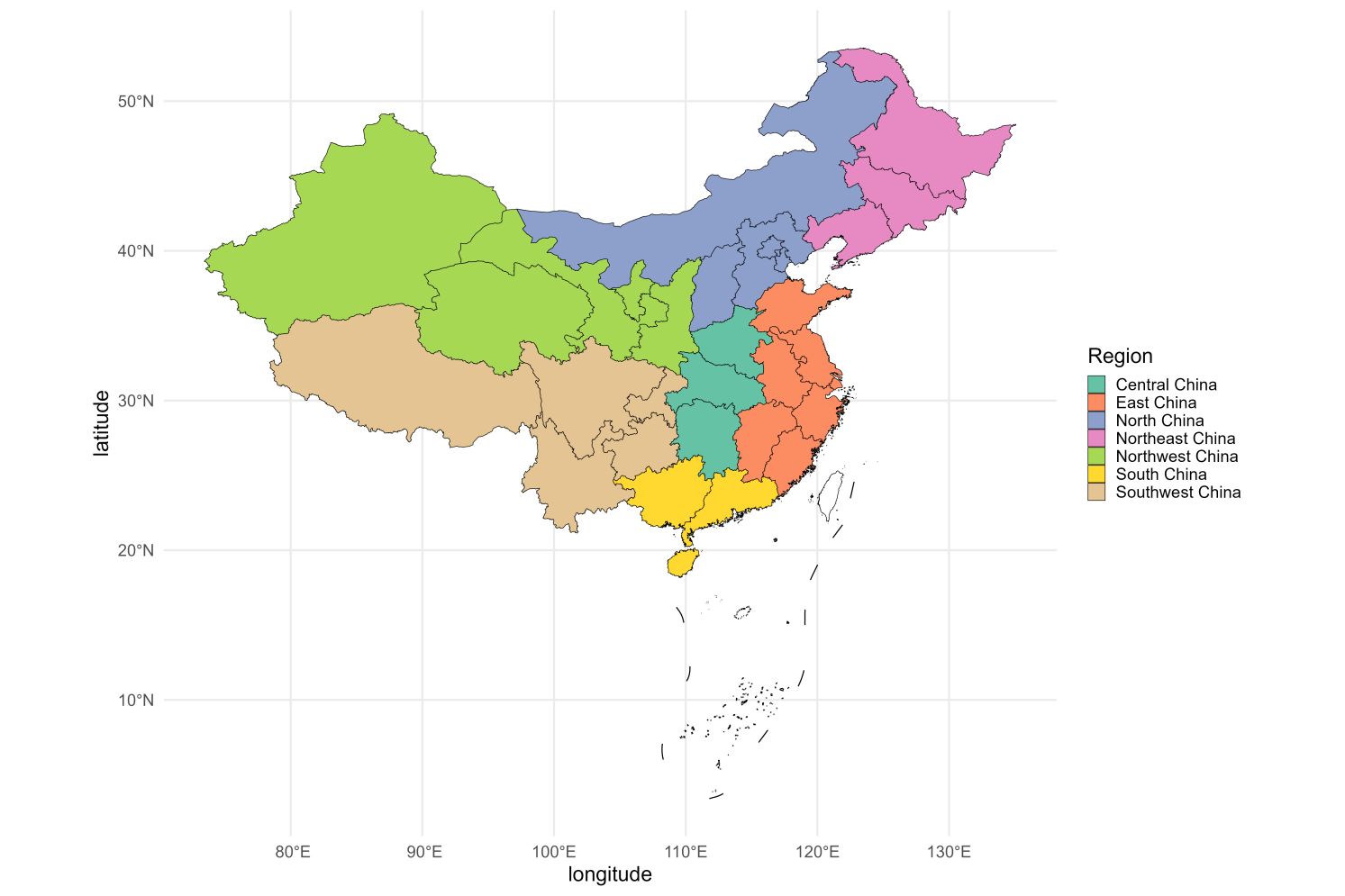


Fig. S1 Distribution of the seven regions in China


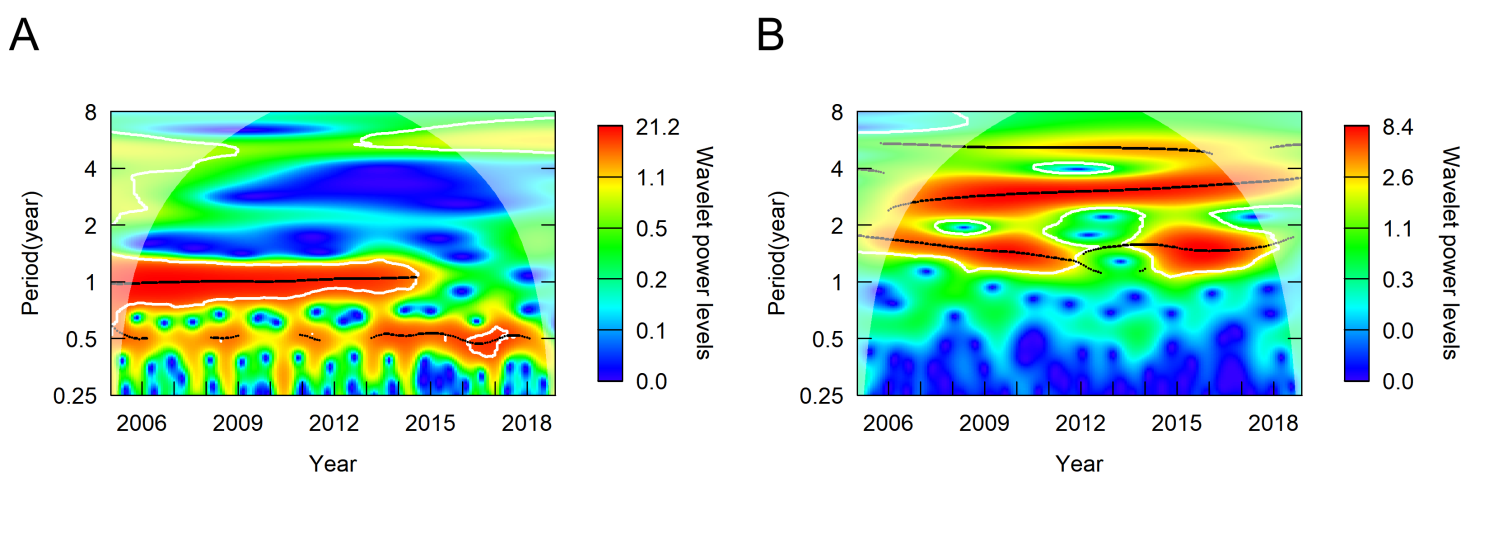


Fig. S2 Wavelet spectra of of OID (A) and ENSO (B) in overall China, 2005–2019.


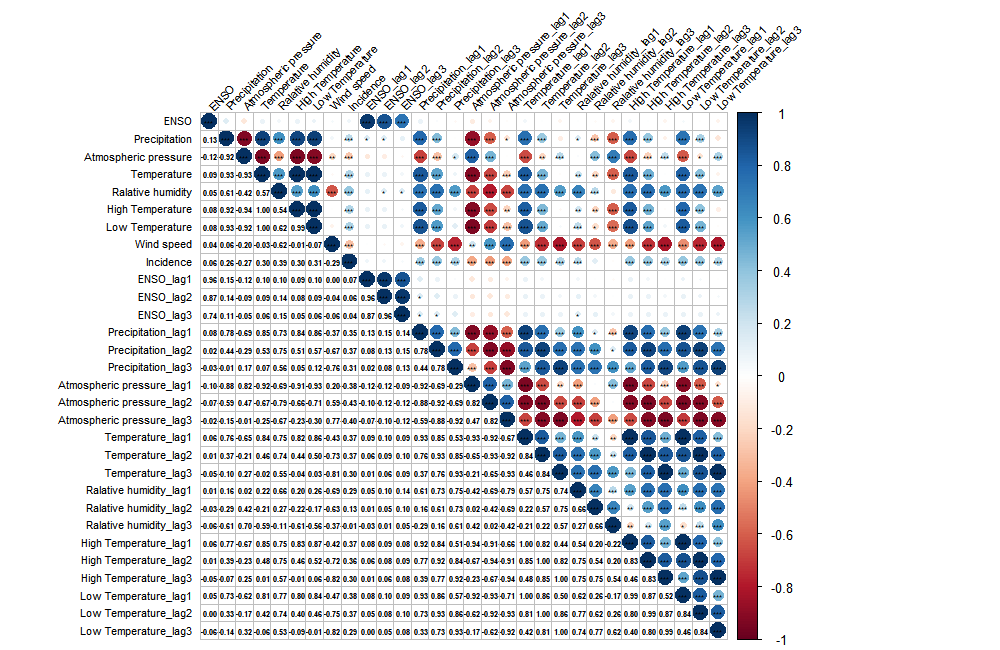


**Fig. S3 Associations between climatic factors and the incidence of OID (***:p<0.001,**:p<0.01,*:p<0.05)**


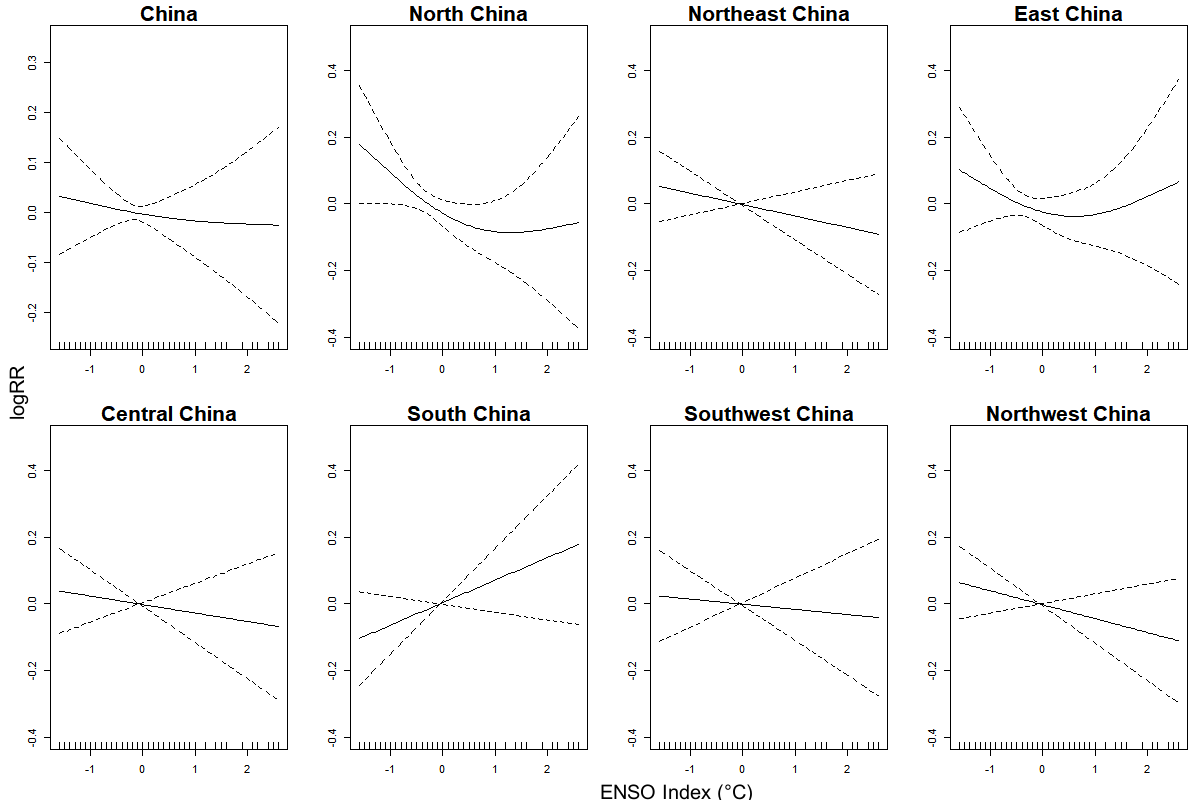


**Fig. S4**  **The exposure-response curves between ENSO index and the incidence of OID in overall China and its seven regions, 2005–2019. (df=4)**


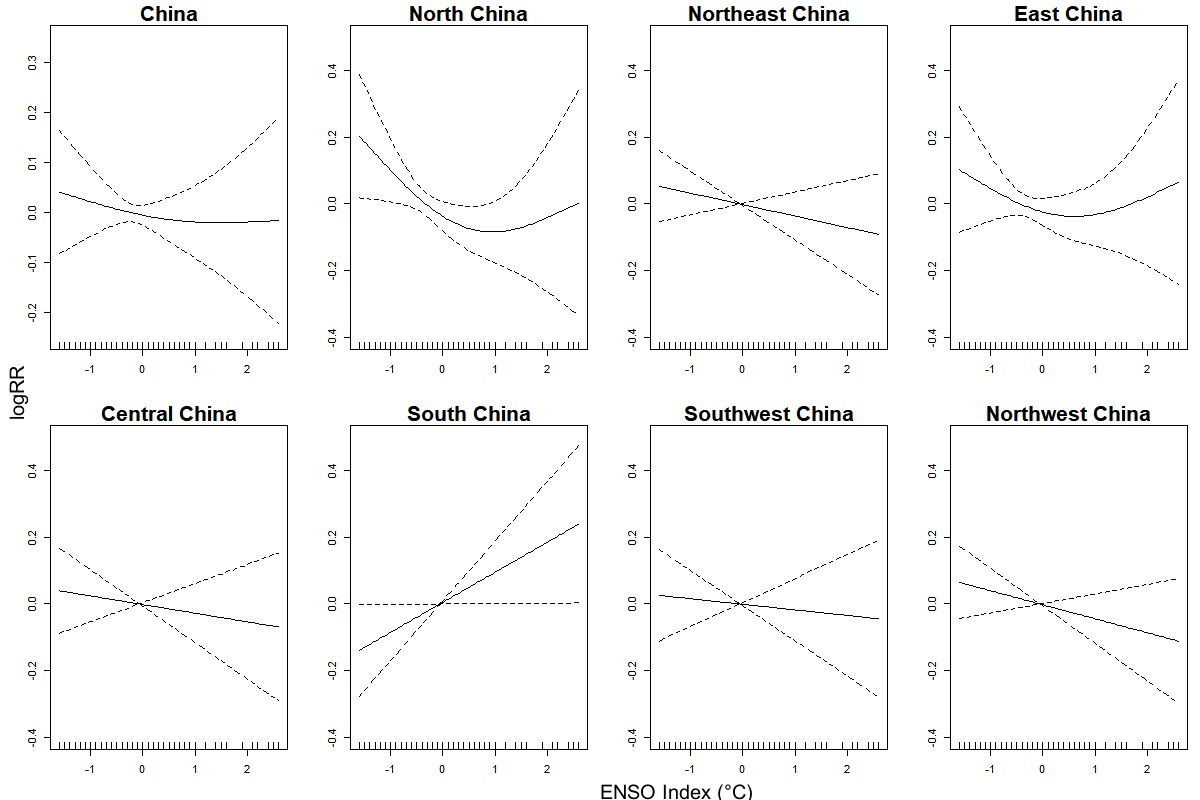


**Fig. S5 The exposure-response curves between ENSO index and the incidence of OID in overall China and its seven regions, 2005–2019. (df=5)**
